# Influence of chronic medical conditions on older patients’ willingness to deprescribe medications: a cross-sectional study

**DOI:** 10.1186/s12877-024-04891-9

**Published:** 2024-04-04

**Authors:** Anabela Pereira, Manuel Veríssimo, Oscar Ribeiro

**Affiliations:** 1 Centre for Health Technology and Services Research, Associate Laboratory RISE– Health Research Network (CINTESIS@RISE), Department of Education and Psychology, University of Aveiro, Campus Universitário de Santiago, 3810-193 Aveiro, Portugal; 2 Institute of Biomedical Sciences Abel Salazar, University of Porto, Rua de Jorge Viterbo Ferreira, 228, 4050-313 Porto, Portugal; 3 Coimbra Institute for Clinical and Biomedical Research (iCBR), Faculty of Medicine, University of Coimbra, Azinhaga de Santa Comba, 3000-548 Coimbra, Portugal

**Keywords:** Deprescribing, Older adults, Patients’ attitudes, Chronic conditions, Multimorbidity

## Abstract

**Background:**

Aging correlates with a heightened prevalence of chronic diseases, resulting in multimorbidity affecting 60% of those aged 65 or older. Multimorbidity often leads to polypharmacy, elevating the risk of potentially inappropriate medication (PIM) use and adverse health outcomes. To address these issues, deprescribing has emerged as a patient-centered approach that considers patients’ beliefs and attitudes toward medication and reduces inappropriate polypharmacy in older adults. Our study aims to investigate whether certain chronic medical conditions are associated with older patients’ willingness to deprescribe medications.

**Methods:**

A cross-sectional study enrolled 192 community-dwelling individuals aged 65 or older taking at least one regular medication. Data included demographics, clinical characteristics, and responses to the Portuguese revised Patients’ Attitudes Towards Deprescribing (rPATD) questionnaire. Descriptive statistics characterized participants, while multiple binary logistic regression identified associations between chronic medical conditions and willingness to deprescribe.

**Results:**

Among the participants (median age: 72 years, 65.6% female), 91.6% had multimorbidity. The analysis revealed that willingness to deprescribe significantly increased with the presence of gastric disease (adjusted odds ratio [aOR] = 4.123; 95% CI 1.221, 13.915) and age (aOR = 1.121; 95% CI 1.009, 1.246). Conversely, prostatic pathology (aOR = 0.266; 95% CI 0.077, 0.916), higher scores in the rPATD appropriateness factor (aOR = 0.384; 95% CI 0.190, 0.773), and rPATD concerns about stopping factor (aOR = 0.450; 95% CI 0.229, 0.883) diminished patients’ willingness to deprescribe.

**Conclusions:**

This study highlights the intricate relationship between older patients’ attitudes toward deprescribing and chronic medical conditions. We found that gastric disease was associated with an increased willingness to deprescribe medications, while prostate disease was associated with the opposite effect. Future research should explore how patients with specific diseases or groups of diseases perceive deprescribing of medications general and for specific medications, aiding in the development of targeted interventions.

**Supplementary Information:**

The online version contains supplementary material available at 10.1186/s12877-024-04891-9.

## Introduction

During the last century and up to the present day, significant improvements in socioeconomic conditions, medical science, and access to medical care have increased life expectancy. Greater longevity has led to a substantial increase in the number of older people. In the 27 countries of the European Union (EU-27), the proportion of older people (i.e., aged 65 and over) in the total population was 20.8% in 2021 [1] and is projected to increase significantly over the coming decades, rising from 90.5 million in 2019 to 129.8 million by 2050 [2].

Aging is associated with an increased prevalence of chronic medical conditions and multimorbidity [3]. The definition of multimorbidity varies in the literature, with some studies defining it as the presence of at least two chronic medical conditions and others as three or more. Despite this variability, the prevalence of multimorbidity has been increasing over time [3, 4] and currently affects 65% of older adults aged 65–84 years and 82% of those aged ≥ 85 years [5].. Additionally, multimorbidity is strongly associated with polypharmacy (concurrent intake of five or more medications per day) [6–9]. Polypharmacy is common among older adults, presenting an overall prevalence of 45% [10]. In Europe, polypharmacy in older adults has reported rates ranging from 26.3% in Switzerland to 39.9% in Portugal [11]; and in the USA, it has been reported as high as 65% [12].

Polypharmacy may be appropriate in cases of multimorbidity, which is frequent in older adults when prescribing multiple medications is often needed and guided by multiple single-disease guidelines [13]. However, it is crucial to acknowledge that the confluence of polypharmacy and older age may lead to a synergistic effect, potentially escalating the risk of adverse drug events (ADEs) and potentially inappropriate medication (PIM) use [14, 15]. The pharmacokinetic and pharmacodynamic changes with age, combined with multimorbidity and polypharmacy, favor drug–drug and drug–disease interactions and increase adverse drug events. In addition, the aging process is associated with a decline in renal and hepatic functions, reduced lean body mass, sensory loss (hearing, vision, and balance), reduced mobility, and cognitive decline, contributing to the adverse outcomes of multiple medication intake [16]. Polypharmacy is independently associated with adverse outcomes in older patients, such as an increased risk of falls, frailty, cognitive impairment, functional decline, hospitalization, and death [14, 17–19]. It has also been strongly associated with specific chronic medical conditions, such as hypertension, diabetes mellitus, chronic kidney disease, and cardiovascular diseases [7].

*Deprescribing* is a patient-centered process to address inappropriate polypharmacy that consists of withdrawal (or dose reduction) of an inappropriate medication supervised by a healthcare professional [20]. This approach fosters shared decision-making between clinicians and patients and has been proven safe and efficient in reducing inappropriate medicines [21, 22]. Deprescribing has been shown to decrease the number of PIM [22–25] and to reduce the incidence of severe adverse drug reactions and falls [18, 22]. However, the impact of deprescribing interventions on mortality remains uncertain [22]. While some studies suggest a reduction in mortality associated with deprescribing, others fail to demonstrate such an effect [21, 23].

Recognizing and considering patients’ beliefs and attitudes towards their medicines is crucial to optimize the shared decision-making process between clinician and patient, which is necessary to increase patient involvement and thus contribute to the success of deprescribing [26, 27]. A recent meta-analysis of studies using the Patients’ Attitudes Towards Deprescribing questionnaire and its revised version (PATD and rPATD, respectively) found that most older patients (84%) were willing to deprescribe [28]. However, a majority (67 to 93%) were still satisfied with their medicines [28]. Similar results were found in a study with Portuguese older patients, in which 83.3% were willing to deprescribe, and 90.6% were satisfied with their current medicines [29]. In addition, two rPATD factors—the *concerns about stopping medications factor* [29–32] and the *appropriateness of medicines* factor [30, 32]—have been associated with patients’ willingness to deprescribe.

Some patients’ characteristics have also been associated with their willingness to have medications deprescribed. These include age [31, 33–36], the number of chronic medical conditions [35, 37, 38], the number of medications [31, 35, 37, 39], self-reported health status [37], and trust in the physician [40]. Another study found a significant association between a specific medical condition, coronary artery disease, and the patient’s willingness to have medications deprescribed [41].

The available evidence also suggests that patients’ willingness to have medications deprescribed differs according to the specific class of medication. For example, in Green et al.’s study (2021) on the association of willingness to deprescribe with health outcome priorities among US older patients, most patients were willing to stop preventive medicines instead of symptom-relieving medications, and they may have been unwilling to stop a particular medicine they considered essential. In addition, statins have been considered less appropriate than other cardiometabolic medications, such as antihypertensives and insulins [42]. Furthermore, older patients were reportedly more willing to have a statin deprescribed than other medicines [43]. Depending on the medicine, these different patient perceptions and attitudes toward deprescribing may arise from several factors, including the perceived severity of the underlying disease [42]. According to a qualitative study conducted among African American men, hypertension was perceived to be more severe than hyperlipidemia [44]. This perception of illness severity may elucidate why older patients, as reported in another study, prioritize antihypertensive medications over statins [42]. Thus, one can hypothesize whether the willingness to have a medication deprescribed is influenced differently by specific medical conditions. This study explores the odds of such an association in a sample of Portuguese older patients.

## Methods

### Study design and population

This study is a pre-planned secondary analysis of a cross-sectional study conducted with older patients recruited from October to December 2021 by convenience sampling from three Portuguese outpatient clinics located in three different cities: Aveiro Coimbra and Viseu. Participants’ inclusion criteria were being aged 65 years or older and taking at least one regular medication. The exclusion criteria were being unable to read Portuguese and having a history of moderate or severe cognitive impairment. A sample size of 192 was appropriate for the exploratory factor analysis of the Portuguese rPATD questionnaire’s validation study [45]; likewise, we considered this sample size sufficient and feasible for the aims of this study.”

#### Data collection

The participants in this study were selected and recruited via convenience sampling during routine medical consultations conducted by a researcher with a medical degree. Informed written consent was obtained from each patient who agreed to participate., The physician-researcher collected data via face-to-face interviews, and the rPATD questionnaire was either self-administered or administered through face-to-face interviews at the participants’ request. The study collected demographic and clinical characteristics of the patients, including age, sex, marital status, residence, education level, involvement in medication management, number of medical appointments in the last 12 months, regular medications, and chronic medical conditions. Likert scales (1 to 5) were used to assess self-reported health status (*bad*, *reasonable*, *good*, *very good*, or *excellent*) and trust in the physician (*very low*, *low*, *medium*, *high*, or *very high*) to evaluate the patient’s trust in their primary care physician. The Charlson age-combined comorbidity index [46] was calculated based on the patient’s age and medical conditions, identified by reconciling the patient’s self-reporting and available medical records.

#### rPATD questionnaire

The Portuguese rPATD questionnaire (older adults version), previously cross-culturally adapted and validated to European Portuguese [45] from its original version [47], was used to assess patients’ attitudes and beliefs about their medication. The rPATD includes 22 questions and consists of two global questions and four factors, with five questions in each factor. The four factors are (1) appropriateness of medication, (2) burden of medication, (3) concerns about stopping medicines, and (4) involvement in medication management. The two global questions are “If my doctor said it was possible, I would be willing to stop one or more of my regular medicines” and “Overall, I am satisfied with my current medicines.” All four-factor questions have a 5-point Likert response scale, from *strongly disagree* = 1 to *strongly agree* = 5, except questions on the rPATD appropriateness factor, which were reverse-scored to represent a greater belief in the appropriateness of their medications. There is no global score; each factor is scored separately, and the two global questions are independently scored, as recommended by the authors of the original scale [47].

#### Age-adjusted Charlson Comorbidity Index

The Charlson Comorbidity Index (CCI) total score relates to predicting 1- and 10-year mortality [48]. In the age-adjusted Charlson Comorbidity Index (age-adjusted CCI), the total score increases by 1 for each additional decade over 40, combining comorbidity burden and age in a single index [46]. The maximum age-adjusted CCI is 37 points.

### Outcomes and statistical analysis

The primary outcome was the chronic medical conditions most associated with older patients’ willingness to have a medication deprescribed. The secondary outcome was the association between the older patients’ willingness to have a medication deprescribed either with the number of chronic medical conditions or with the Charlson age-combined comorbidity index. The “willingness to have a medication deprescribed” was measured by one of the two rPATD global questions: “If my doctor said it was possible, I would be willing to stop one or more of my regular medicines.”

The continuous variables, including the number of regular medications and chronic medical conditions, were categorized for descriptive purposes and to further explore associations with patients’ willingness to have a medication deprescribed. The number of regular medications was categorized based on recognized cutoffs for polypharmacy and excessive polypharmacy to ensure consistency with established definitions and comparability with other studies. Three categories were defined as 1–4, 5–9, 10–14 and ≥ 15. Similarly, the number of chronic conditions was divided into four categories: ≤3, 4–5, 6–7, and ≥ 8. This categorization was determined by combining the definition of multimorbidity with the distribution of results for this variable.

Data were entered and analyzed in SPSS IBM SPSS Version 27.0 (IBM Corp., Armonk, NY, USA) and screened for normality using the Shapiro-Wilk and Kolmogorov-Smirnov tests. Participants’ characteristics and rPATD questionnaire responses were reported using descriptive statistics. Missing data in rPATD questionnaire responses and in participants’ characteristics were excluded from the analysis (Tables 1 and 2 and Additional File 2). Categorical variables were reported as frequencies and percentages, and continuous variables were reported as the median and interquartile ranges (IQR). The rPATD questionnaire factor scores were calculated as previously described [49]. The participants’ responses to the rPATD global question, “If my doctor said it was possible, I would be willing to stop one or more of my regular medicines,” were dichotomized into the binary outcome agree (*strongly agree* or *agree*) or disagree (*unsure*, disagree or *strongly disagree*), hereafter referred to as “willingness to have medications deprescribed.” Multiple binary logistic regression was used to examine whether the patients’ chronic medical conditions were associated with their “willingness to have medications deprescribed.” First, bivariate analyses were performed to assess the association between patients’ willingness to have medications deprescribed and their sociodemographic and clinical characteristics (age, sex, marital status, residence, education level, involvement in medication management, number of medical appointments in the last 12 months, regular medications, number of chronic medical conditions, and age-adjusted CCI), self-reported health status, trust in the physician, and rPATD factor scores. The chi-square (Chi^2^) test was used for categorical variables, and the Mann-Whitney U test was used for continuous and ordinal variables. The variables with a *p*-value ≤ 0.1 were selected to be included as independent variables in the multiple binary logistic regression, and the dependent variable was the dichotomized rPTAD question “willingness to have medications deprescribe.” Then, a multiple binary logistic regression with the backward stepwise likelihood ratio method (backward LR) was performed to verify which of the selected independent variables was a predictor of “willingness to have medications deprescribed.”

A preliminary analysis was performed to verify binary logistic regression assumptions by checking for independent observations, the absence of multicollinearity between the independent variables (all values of tolerance > 0.1 and VF < 10), and whether the continuous predictors were linearly related to a transformed version of the outcome (*p*-value > 0.05). Subsequently, a multiple binary logistic regression was performed using the backward LR method to verify which of the selected independent variables were predictors of “willingness to deprescribe.” The existence of outliers and their influence on the model estimates were then inspected. To isolate points (cases) for which the model fits poorly, studentized residuals (SResid) and deviance statistics were also examined. Specifically, SResid outside 3.3 standard deviations were considered potential outliers, and for deviance statistics, only 5% should lie outside ± 2, and only about 1% should lie outside ± 2.5. Influence statistics leverage was used to examine cases that exerted an undue influence on the model. The leverage cut-off point was three times the average leverage value [3(*k* + 1/*n*)]. The study findings are reported according to the Strengthening the Reporting of Observational Studies in Epidemiology (STROBE) statement checklist [50]; please see Additional File 1 for details.

## Results

A total of 192 participants were included in the study, with a median age of 72 years (IQR = 69–77). Most participants were women (65.6%), and polypharmacy was highly prevalent (76.9%). The majority of participants had a low level of education, with 74.5% having four years or less of education. Despite this, 90.1% of them self-managed their medications. The patients’ sociodemographic and clinical characteristics and rPATD factor scores are presented in Table 1. In terms of chronic medical conditions, multimorbidity (three or more chronic medical conditions) was present in 91.6% of the participants (see Table 2). The age-adjusted CCI median was 9 (IQR = 8–11). The most frequent medical conditions were arthrosis and spinal disorders (97.9%), hypertension (85.4%), dyslipidemia (76.6%), anxiety or panic disorder (42.7%), history of gastric disease (40.6%), overweight or obesity (37.5%), chronic pain (35.4%), depression (34.9%), and peripheral vascular disease (32.8%). Regarding the patients’ attitudes, the rPATD questionnaire revealed that 83.33% were willing to stop one or more of their regular medicines if the doctor said it was possible. The majority (90.62%) were satisfied with their current medicines. The rPATD factor scores are presented in Table 1.

**Table 1:** Patients’ characteristics and attitudes toward deprescribing medications

Characteristics	Values		
**Age (** ***N*** ** = 192)**			
median (IQR)	72 (69–77)		
**Sex (*N*** **= 192)**	*n* (%)		
male	66 (34.4)		
female	126 (65.6)		
**Level of education (*N*** **= 191)**	*n* (%)		
primary school (1 to 4 years)	143 (74.5)		
lower secondary education (5 to 9 years)	28 (14.6)		
higher secondary education (10 to 12 years)	11(5.7)		
university degree or more	9 (4.7)		
**Medication management (*N*** **= 189)**	*n* (%)		
self-management	173 (90.1)		
self-management with the help of a family member or friend	7 (3.6)		
family member or friend	7 (3.6)		
**Number of regular medications (*N*** **= 192)**	value		
median (IQR)	6 (5–9)		
	*n* (%)		
1 to 4	43 (22.4)		
5 to 9	115 (59.9)		
10 to 14	27 (14.1)		
≥ 15	4 (2.1)		
**Trust in the physician (*N*** **= 170)**^**a**^	value		
median (IQR)	4 (3–5)		
**Self-reported health status (*N*** **= 171)**^**b**^	*n* (%)		
bad	20 (10.4)		
reasonable	128 (66.7)		
good, very good, or excellent	30 (15.6)		
**Medical appointments in the last 12 months (*N*** **= 160)**	value		
median (IQR)	5 (3–8)		
**rPATD questionnaire**			
Global question: “If my doctor said it was possible, I would be willing to stop one or more of my regular medicines.” (*N* = 192)		*n* (%)	
- agree (strongly agree, agree)	160 (83.3)		
- disagree (unsure, disagree, strongly disagree)	32 (16.7)		
**rPATD factors scores** ^**c**^	median (IQR)		
Involvement in medication management (*N* = 190)	4.4 (4.0–4.8)		
Burden of medication (*N* = 192)	2.6 (2.0–3.6)		
Appropriateness of medication (*N* = 191)	3.4 (2.6–4.0)		
Concerns about stopping medication (*N* = 192)	3.0 (2.4–3.4)		

Abbreviations: IQR: interquartile range; rPATD: revised Patients’ Attitudes Towards Deprescribing

(a) Trust in the physician was assessed using a Likert scale (1 = *very low*, 2 = *low*, 3 = *medium*, 4 = *high*, 5 = *very high*) to evaluate the patient’s trust in their primary care physician

(b) Self-reported health status was assessed by a Likert scale (1 = bad, 2 = reasonable, 3 = good, 4 = very good, 5 = excellent)

(c) rPATD scores range between 1 and 5, with higher scores indicating a higher perceived burden of medicines, belief in the appropriateness of medicines, concerns about stopping medicines, and involvement in medication management

**Table 2:** Participants’ chronic medical conditions (*N* = 192)

Number of chronic medical conditions	Values
median (IQR)	6 (5–7)
**Number of chronic medical conditions** ^**a**^	*n* (%)
≤ 3 medical conditions	16 (8.3)
4 to 5 medical conditions	58 (30.2)
6 to 7 medical conditions	74 (38.5)
≥ 8 medical conditions	44 (22.9)
**Chronic medical conditions**	*n* (%)
Myocardial infarction (history of)	13 (6.8)
Coronary disease/angina	25 (13)
Congestive heart failure	17 (8.9)
Hypertension	164 (85.4)
Dyslipidemia	147 (76.6)
Peripheral vascular disease	63 (32.8)
Cerebrovascular disease	14 (7.3)
Hemiplegia or paraplegia	9 (4.7)
Other neurologic disease ^b^	11 (5.7)
Chronic pulmonary disease	18 (9.4)
Diabetes without end-organ damage	47 (24.5)
Diabetes with end organ	7 (3.6)
Chronic renal disease (moderate to severe)	3 (1.6)
Mild liver disease	8 (4.2)
Moderate to severe liver disease	1(0.5)
Gastric disease (any history of ulcer disease treatment or prevention, ulcer bleeding, or GERD)	78 (40.6)
Prostate disease (benign hypertrophy, neoplasia)	30 (15.6)
Neoplasia (solid tumor other than prostate)	29 (15.1)
Metastatic solid tumor	2 (1.04)
Lymphoma	4 (2.1)
Arthrosis, spinal disorders, or rheumatoid arthritis	188 (97.9)
Chronic pain	68 (35.4)
Osteoporosis	31 (16.1)
Overweight or obesity	72 (37.5)
Depression	67 (34.9)
Anxiety, panic disorder	82 (42.7)
Other	4 (2.1)
**Age-adjusted Charlson Comorbidity Index**^**c**^ (***N*** **= 192)**	
Median (IQR)	9 (8–11)

Abbreviations: IQR: interquartile range; GERD: gastroesophageal reflux disease

a) Chronic medical conditions divided into categories according to frequency

b) All neurological conditions, excluding dementia, hemiplegia, or paraplegia

c) The age-adjusted Charlson Comorbidity Index (age-adjusted CCI) combines the comorbidity burden measured by the Charlson Comorbidity Index (CCI) and age in a single index, and the total score increases by 1 for each additional decade over 40 [48]. The CCI total score relates to predicting 1- and 10-year mortality [50]. The maximum age-adjusted CCI is 37 points

The variables with a *p*-value < 0.1 in bivariate analysis were sex, medical appointments in the last 12 months, rPATD appropriateness factor, rPATD burden of medication, rPATD concerns about stopping medications factor, chronic pulmonary disease, other neurologic diseases (neurologic conditions, excluding dementia, hemiplegia, or paraplegia), gastric disease (any history of ulcer disease treatment or prevention, ulcer bleeding, or gastroesophageal reflux disease - GERD), prostate disease (benign hypertrophy, neoplasia), and chronic pain. Although lymphoma and dementia presented a *p*-value < 0.1, they were excluded because they had fewer than five cases each. The bivariate analysis results are shown in Additional File 2.

All statistical assumptions were verified except for the variable rPATD appropriateness score, which violated the assumption of linearity between the continuous predictors and the transformed version of the outcome (*p* = 0.030). Nonetheless, as medication appropriateness was considered a clinically relevant variable, it was included in the multiple binary regression. As for the existence of outliers and their influence on the model estimates, the SResid absolute values were all inferior to 3.3; 6.25% of the cases were above 2, and 1.25% presented values above 2.5. The leverage cut-off point was [3(11 + 1/160)] = 0.225; one case was above this value (0.230). These multiple binary regression model results are shown in Additional File 3. Each case presenting SResid above 2 was inspected, and no data entry error or reason for excluding one of them from the analysis was found. Then, a sensitivity analysis was conducted to assess the influence of the case with a leverage value above the cut-off on the model, followed by another multiple binary logistic regression, without that one case, to compare the regression models. This final model (Table 3) was significant [X2 [7] = 34.016; *p* < 0.001], explaining 33.7% of the variance (Nagelkerke R Square) and correctly predicting 84.9% of the results. There was a good model fit (Hosmer-Lemeshow test nonsignificant, *p* = 0.336).

In the first multiple logistic binary regression model, age, gastric disease (or history of), prostatic disease, the rPATD appropriateness factor, and the rPATD concerns about stopping factor were significantly associated with patients’ willingness to have a medication deprescribed. Gastric disease (or history of) was a positive predictor (aOR = 5.217; 95% CI 1.452, 18.740), whereas prostatic disease (aOR = 0.217; 95% CI 0.063, 0.775) was a negative predictor (Additional File 3).

In the multiple logistic binary regression final model, age, gastric disease (or history of), prostatic pathology, the rPATD appropriateness factor, and rPATD concerns about stopping factors were significantly associated with patients’ willingness to have a medication deprescribed. Age (aOR = 1.121; 95% CI 1.009, 1.246) and gastric disease (aOR = 4.123; 95% CI 1.221, 13.915) were positive predictors, whereas prostatic pathology (aOR = 0.266; 95% CI 0.077, 0.916), the rPATD appropriateness factor (aOR = 0.384; 95% CI 0.190, 0.773), and the rPATD concerns about stopping factor (aOR = 0.450; 95% CI 0.229, 0.883) were negative predictors (see Table 3).

**Table 3:** Associations between chronic medical conditions and older patients’ willingness to have medications deprescribed (*N* = 192) ^a^

Variable	*P* value	aOR	95% CI	
			Lower	Upper
Age (years)	0.034	1.121	1.009	1.246
Gastric disease ^c^	0.022	4.123	1.221	13.915
Prostatic disease^d^	0.036	0.266	0.077	0.916
rPATD appropriateness factor	0.007	0.384	0.190	0.773
rPATD concerns about stopping factor	0.020	0.450	0.229	0.883
Constant	0.852	0.473		

aOR: adjusted odds ratio; CI: confidence interval; rPATD: revised Patients’ Attitudes Towards Deprescribing

a) Multiple logistic binary regression with backward LR method. The rPATD global question “If my doctor said it was possible, I would be willing to stop one or more of my regular medicines” was dichotomized to a binary outcome: *agree* (strongly agree or agree) and *disagree* (unsure, disagree, or strongly disagree), designated as “willingness to have medications deprescribed.” As independent variables to include in the multiple binary logistic regression model, we selected the patients’ demographic and clinic characteristics, the independent variables with *p* < 0.100 in bivariate analysis, and the variable age, which was considered clinically relevant

b) All assumptions were satisfied for the selected variables, except for the rPATD appropriateness factor score. However, it was included in the multiple binary regression because it was considered a clinically relevant variable

c) Any history of ulcer disease treatment or prevention, ulcer bleeding, or GERD (gastroesophageal reflux disease)

d) Prostate benign hypertrophy and prostatic neoplasia

## Discussion

This study provides valuable insights into the relationship between specific chronic medical conditions and the willingness of older patients to engage in deprescribing. Two chronic medical conditions, gastric disease (or a history of it) and prostate disease, were associated with older patients’ willingness to deprescribe one or more of their medications if their doctors recommended it.

We highlight that within our study population, 78 participants (40.6%) had a history of gastric disease; of these, an impressive 93.6% expressed a willingness to consider deprescribing one or more medications. The initial bivariate analysis showed a statistically significant association (*p* = 0.002) between gastric disease and the willingness of older patients to deprescribe. Subsequently, in our multiple logistic binary regression, it was established that the odds of older patients being willing to deprescribe one or more of their medicines increased by a factor of 4.123 (aOR = 4.123; 95% CI 1.221, 13.915) if they had gastric disease (or a history of it). This finding suggests that individuals with gastric disease might be hypothetically more disposed to deprescribing medications due to concerns about potential adverse effects. This may be supported by their previous experience or common knowledge, perceiving a reduction in the number of medications as advantageous.

Regarding the second chronic medical condition associated with willingness to deprescribe one or more of their medications, out of 66 male participants, 30 had prostate disease. Among them, 66.7% expressed a willingness to deprescribe one or more medications, a slightly lower proportion than the average response rate of 83.3% observed in the study’s total population. The initial bivariate analysis uncovered a significant correlation (*p* = 0.008) between prostate disease and older patients’ willingness to deprescribe. Furthermore, the multiple binary logistic analysis determined that prostate disease decreased the odds of older patients being willing to deprescribe by a factor of 3.8 (aOR = 0.266; 95% CI 0.077, 0.916). While this study utilized the rPATD questionnaire to assess participants’ willingness to deprescribe medications if the doctor recommended them without specifying drug classes, it is plausible that patients ascribe greater importance to prostate medications than others, influencing the observed results. An investigation examining attitudes toward deprescribing alpha-blockers for male lower urinary tract symptoms (LUTS), with specific questions about alpha-blockers added to the rPATD questionnaire, found that 93% of participants were willing to deprescribe medications at their doctor’s request [50]. However, in the same study, a lower percentage (61%) were willing to participate in the proposed alpha-blocker discontinuation trial, suggesting that patients with LUTS attribute importance to these medications. Thus, while this study did not directly inquire about prostate medication deprescribing, the observed decrease in the odds of being willing to deprescribe medications indicates that participants with this condition may have had prostate drugs in mind when answering the question. Furthermore, previous studies have indicated that patients with benign prostatic hypertrophy (BPH) are chiefly worried about the possibility of serious urological complications and the need for surgical intervention. This type of intervention entails risks both during and after the procedure, such as urinary incontinence or ejaculatory dysfunction [51–54]. As a result, most of BPH patients expect to receive pharmacological treatment to help mitigate these risks [52, 53]. Our study’s results align with this finding, as patients with prostatic disease have a lower likelihood of being willing to deprescribe.

The associations between gastric disease (or history of it) and prostate disease and older patients’ willingness to deprescribe medications were statistically significant. Nevertheless, the wide confidence intervals indicate some uncertainty in the precise estimated odds ratios, potentially necessitating a larger sample size to enhance the precision of these estimates. Although the confidence intervals are wide, it appears that patients with gastric disease are more likely to participate in deprescribing interventions if recommended by their doctor. In contrast, prostate disease has the opposite effect. These findings likely reflect patients’ beliefs regarding the impact of medications on underlying medical conditions.

To the best of our knowledge, this study is the first to identify specific chronic medical conditions as predictors of the willingness to deprescribe medications among older patients. Prior research involving 300 community-dwelling older Lebanese patients identified a significant association between coronary artery disease and older patients’ willingness to deprescribe medications in an initial bivariate analysis; however, this association did not persist in further multivariate analysis [41]. Another study found that willingness to deprescribe declined as frailty risk scores increased [55]. However, it included hospitalized patients, not community-dwelling individuals, and used a risk score rather than specific chronic medical conditions as the independent variable.

This study’s multiple logistic binary regression results also revealed that age, rPATD concerns about stopping medications, and rPATD appropriateness factor scores were associated with older patients’ willingness to deprescribe one or more medications. Given its association with increased multimorbidity, polypharmacy, and rPATD factors, we included age as an independent variable in the multiple logistic regression due to its clinical relevance. Our analysis confirmed our initial assumptions, showing that older age increased the odds of being willing to deprescribe medications by a factor of 1.121. This suggests that the probability of older patients being willing to deprescribe medications increases with each additional year of life. The rPATD appropriateness factor (aOR = 0.384) and rPATD concerns about stopping medications factor (aOR = 0.450) significantly reduced the odds of older patients’ willingness to deprescribe a medication. Specifically, a one-point increase in the rPATD appropriateness factor reduced the odds by a factor of 2.6, indicating that patients’ beliefs about the appropriateness of their medications may hinder their willingness to participate in deprescribing interventions. These results align with previous research findings [30, 32, 41, 56]. Additionally, these findings indicate that a one-point increase in rPATD concerns about stopping factor reduces the odds of older patients’ willingness to deprescribe medications by more than half. These results are also consistent with previous studies evaluating attitudes toward deprescribing, which identified the rPATD “concerns about stopping” factor as a negative predictor of patients’ willingness to deprescribe [30–57, 41, 58, 59].

## Strengths and limitations

This study represents the first attempt to explore the influence of chronic medical conditions on older patients’ willingness to have medications deprescribed. As such, it fills a significant gap in the existing literature, shedding light on an underresearched area of clinical practice. To assess older patients’ attitudes toward their medication, the study used a questionnaire (rPATD) previously translated and validated for the Portuguese older population. This questionnaire has been widely utilized in international research, enhancing the comparability of findings with those from other regions. The identification of chronic medical conditions in the study was achieved through a comprehensive data collection process. This involved patient self-reporting and data collection by a medical investigator who had access to information provided by healthcare professionals. This multifaceted approach aimed to minimize the inaccuracies associated with patient self-reports and the absence of access to clinical data from the national healthcare service. In addition, the use of the rPATD questionnaire, which may be susceptible to desirability bias inherent in self-report instruments, was mitigated by the overall study design, as the researchers were not directly involved in patient care, reducing the potential for participants to overreport their willingness to accept deprescribing. This approach bolsters the reliability of the collected data.

A significant limitation is the convenience sampling used in this study, which may not accurately represent the broader population of older patients, introduce selection bias, and affect the generalizability of our results. Furthermore, the sample size may have impacted the results obtained. While statistical significance was achieved, it is noteworthy that the confidence intervals were wide, indicating substantial variability. A larger sample might have provided more precise estimates. Therefore, caution should be exercised when interpreting point estimates. Also important is that the potential for desirability bias, common to all self-administered questionnaires, cannot be eliminated entirely. Although the study design aimed to minimize this bias, as the researchers were not directly involved in patient care, it reduced the potential for participants to overreport their willingness to accept deprescription. This approach bolsters the reliability of the collected data. The rPATD questionnaire, designed for quantitative research, provided valuable insights into patient attitudes. However, its structured nature limited the ability to conduct an in-depth investigation into the nuances of patients’ attitudes toward medication.

In addressing the limitations of our study, it is essential to underscore the need for future investigations into the combined influence of chronic diseases and their corresponding medications on patients’ attitudes toward deprescribing. While our research utilized the Revised Patients’ Attitudes Toward Deprescribing questionnaire, rigorously translated, and validated for Portuguese adults, it primarily assesses patients’ overall attitudes toward medications rather than toward specific therapeutic classes. An area for future research lies in adapting the Portuguese version of this questionnaire to include tailored questions for specific medications, offering a nuanced understanding of patients’ deprescribing attitudes. Patients’ beliefs about their medicines and medication adherence are intricately linked to perceptions of illness severity and treatment efficacy, balancing potential benefits against risks of adverse events. Our results emphasize the importance of exploring the relationship between chronic medical conditions and older patients’ attitudes toward deprescribing. Recognizing the multifaceted nature of patients’ attitudes and beliefs regarding their medication is crucial, where concerns about specific drugs may play a significant role. Therefore, it is imperative to advance our understanding of how chronic diseases and their associated medications influence patients’ attitudes toward deprescribing. This effort will aid in tailoring deprescribing discussions to align with patients’ beliefs and attitudes, fostering shared decision-making, and contributing to deprescribing success.

Our study utilized the Age-adjusted Charlson Comorbidity Index, a comprehensive tool that combines comorbidity burden and age into a single index, to identify chronic conditions. However, it should be noted that certain chronic conditions, such as insomnia and other sleep disturbances, were not included in our analysis as they are not part of the chosen index. These conditions are frequently linked with benzodiazepine use, which patients may be hesitant to deprescribe. Future studies incorporating these chronic conditions may offer a more thorough comprehension of patients’ attitudes toward their medications.

Another possible limitation to consider is the low level of education of the participants, which tends to be associated with low levels of health literacy. According to our study results, a significant majority of participants (74.5%) had completed four years or less of primary school education. Although this may appear to be a high figure, it is in line with current educational statistics in Portugal, albeit slightly higher. As of 2022, around 21.4% of the population had completed primary education (i.e., the first cycle of basic education lasting four years). Specifically, within the age group of our study participants (i.e., individuals aged 65 and older), 59.43% had four years of education in 2022 [60, 61]. It is worth noting that the participants in our study were recruited from healthcare settings. Individuals with lower levels of education are more likely to experience health problems and, therefore, seek healthcare services more frequently [62]. This may be one of the factors that contribute to the observed difference.

In summary, while this study contributes to our understanding of older patients’ willingness to undergo deprescription in the context of chronic medical conditions, it is essential to recognize that future research could build upon these findings to explore the observed associations in greater detail, potentially employing mixed methods approaches to gain deeper insights into patient attitudes.

## Conclusions

This study highlights the intricate interplay between chronic medical conditions and older patients’ attitudes toward deprescribing. The findings indicated that gastric disease (or a history of it) was associated with increased older patients’ willingness to stop taking a medication if their doctor recommended it. Conversely, prostate disease decreases the odds of older patients being willing to deprescribe. In addition, greater concerns about stopping medication and higher beliefs in the appropriateness of their medication were found to be associated with a decrease in older patients’ willingness to deprescribe, whereas age increased this willingness. In pursuing patient-centered care, understanding and addressing the multifaceted factors influencing attitudes toward deprescribing among older patients are paramount, and this study’s findings provide valuable insights in this direction. Future research and clinical practice should continue to embrace the significance of these factors to refine deprescribing strategies and enhance the overall medication appropriateness and quality of care for older patients. Additionally, exploring how chronic medical conditions influence patients’ attitudes toward the medications associated with them and toward medicines in general will aid in developing targeted interventions.

### Electronic supplementary material

Below is the link to the electronic supplementary material.

Supplementary Material 1

Supplementary Material 2

Supplementary Material 3

## Data Availability

The data that support this study’s findings are available upon request from the corresponding author.
