# Supplementary material for: Influence of chronic medical conditions on older patients’ willingness to deprescribe medications: a cross-sectional study
Source: BMC Geriatr. 2024 Apr 4;24:315. doi: 10.1186/s12877-024-04891-9 (PMC10993447; doi:10.1186/s12877-024-04891-9)
Supplement: Supplementary file 2 — Supplementary Material 2 [file 12877_2024_4891_MOESM2_ESM.docx]

**Additional File 2**

Table A2*:* Patients Characteristics and rPATD Factors Scores for the Study´s Total Population and Stratified by “*Willingness to have medications Deprescribed.” ^a^*

| **Characteristic** | **Total** | **Willingness to have medications deprescribed** | | *bivariate analyses* |
| --- | --- | --- | --- | --- |
|  | value | **disagree** | **agree** |  |
| **Age (N = 192)** |  |  |  | *p* value ^b^ |
| median (IQR) | 72 (69-77) | 71.5 (68-76) | 73 (69-77.75) | *p*= 0.193 |
| **Sex (N = 192)** | n (%) | n (%) | n (%) |  |
| male | 66 (34.4) | 16 (50) | 50 (31.3) | ***p*=0.041** |
| female | 126 (65.6) | 16 (50) | 110 (68.8) |  |
| **Marital Status (N=183)** | n (%) | n (%) | n (%) |  |
| married | 127 (66.1) | 20 (64.5) | 107 (66.9) | *p*=0.756 |
| widower | 33 (17.2) | 7 (22.6) | 26 (16.3) |  |
| other | 23 (12) | 4 (12.9) | 19 (11.9) |  |
| **Residence (N=184)** | n (%) | n (%) | n (%) |  |
| own or rented house | 177 (96.2) | 30 (96.8) | 147 (96.1) | *p*=0.802 |
| family house | 5 (2.7) | 1 (3.1) | 4 (2.6) |  |
| institution / nursing home | 2 (1.1) | 0 (0) | 2 (1.3) |  |
| **Resides with: (N=183)** | n | n (%) | n (%) |  |
| alone | 39 (21.3) | 8 (25.8) | 31 (20.4) | *p*=0.727 |
| spouse or companion | 99 (54.1) | 16 (51.6) | 83 (54.6) |  |
| relatives | 14 (7.7) | 1 (31.2) | 13 (8.6) |  |
| spouse and relatives | 29 (15.8) | 6 (19.4)) | 23 (15.1) |  |
| other | 2 (1.9) | 0 (0) | 2 (1.3) |  |
| **Level of education (N= 191)** | n (%) | n (%) | n (%) |  |
| primary school (1 to 4 years) | 143 (74.5) | 26 (81.3) | 117 (73.6) | *p*=0.660 |
| lower secondary education (5 to 9 years) | 28 (14.6) | 3 (9.4) | 25 (15.7) |  |
| higher secondary education (10 to 12 years) | 11(5.7) | 1 (3.1) | 10 (6,3) |  |
| university degree or more | 9 (4.7) | 2 (6.3) | 7 (4,4) |  |
| **Medication Management (N=189)** | n (%) | n (%) | n (%) |  |
| self-management | 173 (90.1) | 29 (93.5) | 144 (92.3) | *p*= 0.232 |
| self-management with the help of a family member or friend | 7 (3.6) | 1 (3.2) | 6 (3.8) |  |
| family member or friend | 7 (3.6) | 1 (3.2) | 6 (3.8) |  |
| **Number of regular medications (N=192)** | value | value | value |  |
| median (IQR) | 6 (5-9) | 5 (4-7) | 6 (4-8) | *p*= 0.589 |
|  | n (%) | n (%) | n (%) |  |
| 1 to 4 | 43 (22.4) | 8 (25.8) | 35 (22.2) | *p*= 0.447 |
| 5 to 9 | 115 (59.9) | 19 (61.3) | 96 (60.8) |  |
| 10 to 14 | 27 (14.1) | 2 (6.5) | 25 (15.8) |  |
| ≥ 15 | 4 (2.1) | 2 (6.5) | 2 (1.3) |  |
| **Trust in the Physician (N= 170) ^c^** | value | value | value |  |
| median (IQR) | 4 (3-5) | 4 (3.25-5) | 4 (3-5) | *p*=0.649 |
| **Self-reported Health Status (N=171) ^d^** | n (%) | n (%) | n (%) |  |
| bad | 20 (10.4) | 5 (17.2) | 15 (10.1) | *p*=0,390 |
| reasonable | 128 (66.7) | 18 (62.1) | 110 (73.8) |  |
| good, very good or excellent | 30 (15.6) | 6 (20.7) | 24 (16.1) |  |
| **Medical Appointments in the last 12 months (N=160)** | value | value | value |  |
| median (IQR) | 5 (3-8) | 4 (2.5-6.5) | 5 (4-8) | ***p*=0.025** |
| **rPATD factors scores ^e^** |  |  |  |  |
| median (IQR) | value | value | value |  |
| Involvement (N=190) | 4.4 (4.0 -4.8) | 4.4 (4.0 -4.8) | 4.2 (4.0 -4.8) | *p*= 0.400 |
| Burden (N=192) | 2.6 (2.0- 3.6) | 2.4 (1.8-2.8) | 2.8 (2.0-3.6) | *p* =0.065 |
| Appropriateness (N=191) | 3.4 (2.6-4.0) | 4 (2.85-4.35) | 3.4 (2.6-4.0) | ***p* =0.033** |
| Concerns about stopping (N=192) | 3.0 (2.4 -3.4) | 3.4 (3.05- 3.8) | 2.8 (2.20-3.4) | ***p* < 0.001** |
| **Chronic medical conditions (N=192)** |  |  |  |  |
|  | n (%) | n (%) | n (%) |  |
| ≤ 3 medical conditions | 16 (8.3) | 5 (15.6) | 11 (6.9) | *p*= 0. 492 |
| 4 to 5 medical conditions | 58 (30.2) | 9 (28.1) | 49 (30.6) |  |
| 6 to 7 medical conditions | 74 (38.5) | 12 (37.5) | 62 (38.8) |  |
| ≥ 8 medical conditions | 44 (22.9) | 6 (18.8) | 38 (23.8) |  |
|  |  | Willingness to have medications deprescribed | | *bivariate analyses* |
| **Chronic medical conditions (N=192)** | n (%) | disagree | agree | *p* value ^b^ |
| Myocardial infarction (history of) | 13 (6.8) | 1 (7.7) | 12 (92.3) | 0.369 |
| Coronary disease/angina | 25 (13) | 3 (12) | 22 (88) | 0.502 |
| Congestive heart failure | 17 (8.9) | 1 (5.9) | 16 (94.1) | 0.211 |
| Hypertension | 164 (85.4) | 27 (16.5) | 137 (83.5) | 0.855 |
| Dyslipidemia | 147 (76.6) | 24 (16.3) | 123 (83.7) | 0.819 |
| Chronic pulmonary disease | 18 (9.4) | 6 (33.3) | 12 (66.7) | **0.046** |
| Peripheral vascular disease | 63 (32.8) | 9 (14.3) | 54 (85.7) | 0.536 |
| Cerebrovascular disease | 14 (7.3) | 3 (21.4) | 11 (78.6) | 0.620 |
| Dementia | 3 (1.6) | 2 (66.7) | 1 (33.3) | 0.019 |
| Hemiplegia | 9 (4.7) | 3 (33.3) | 6 (66.7) | 0.169 |
| Other neurologic disease | 11 (5.7) | 3 (27.3) | 8 (72.7) | **0.071** |
| Diabetes without end organ damage | 47 (24.5) | 10 (21.3) | 37 (78.7) | 0.329 |
| Diabetes with end organ | 7 (3.6) | 0 (0) | 7 (100) | 0.228 |
| Chronic renal disease (moderate to severe) | 3 (1.6) | 0 (0) | 3 (100) | 0.435 |
| Mild liver disease | 8 (4.2) | 0 (0) | 8 (100) | 0.196 |
| Moderate to severe liver disease | 1(0.5) | 0 (0) | 1 (100) | 0.654 |
| Gastric disease (gastritis, peptic disease ulcer, GERD) | 78 (40.6) | 5 (6.4) | 73 (93.6) | **0.002** |
| Prostate disease (benign hypertrophy, neoplasia) | 30 (15.6) | 10 (33.3) | 20 (66.7) | **0.008** |
| Neoplasia (solid tumor other than prostate) | 29 (15.1) | 4 (13.8) | 25 (86.2) | 0.652 |
| Metastatic solid tumor | 2 (1.04) | 0 (0) | 2 (100) | 0.204 |
| Lymphoma | 4 (2.1) | 2 (50) | 2 (50) | 0.071 |
| Arthrosis, spinal disorders, or rheumatoid arthritis | 188 (97.9) | 31(16.5) | 157 (83.5) | 0.651 |
| Chronic pain | 68 (35.4) | 7 (10.3) | 61(89.7) | **0.079** |
| Osteoporosis | 31 (16.1) | 7 (22.6) | 24 (77.4) | 0.335 |
| Overweight or obesity | 72 (37.5) | 13 (18) | 59 (82) | 0.689 |
| Depression | 67 (34.9) | 10 (14.9) | 57 (85.1) | 0.636 |
| Anxiety, panic disorder | 82 (42.7) | 17 (20.7) | 65 (79.3) | 0.192 |
| Connective tissue disease | 1 (0.5) | 0 (0 ) | 1 (100) | 0.654 |

Abbreviations: IQR, interquartile range; rPATD, revised Patients’ Attitudes Towards Deprescribing; GERD, gastroesophageal reflux disease.

(a) The rPATD global question "If my doctor said it was possible, I would be willing to stop one or more of my regular medicines" were dichotomized into the binary outcome agree (strongly agree or agree) or disagree (unsure, disagree or strongly disagree), hereafter referred to as “willingness to have medications deprescribed.”

(b) *p-*value derived from the Mann–Whitney U and Chi-square tests in those presented as median (IQR) and n (percentage), respectively.

(c) Trust in the physician was assessed by a Likert scale (1=very low, 2=low, 3=medium, 4=high, 5=very high).

(d) Self-reported health status assessed by a Likert scale (1=bad, 2=reasonable, 3=good, 4=very good, 5=excellent)

(e) rPATD scores range between 1 and 5, with higher scores indicating a higher perceived burden of medicines, belief in the appropriateness of medicines, concerns about stopping medicines, and involvement in medication management.
