# Supplementary material for: Influence of chronic medical conditions on older patients’ willingness to deprescribe medications: a cross-sectional study
Source: BMC Geriatr. 2024 Apr 4;24:315. doi: 10.1186/s12877-024-04891-9 (PMC10993447; doi:10.1186/s12877-024-04891-9)
Supplement: Supplementary file 3 — Supplementary Material 3 [file 12877_2024_4891_MOESM3_ESM.docx]

**Additional File 3**

Table A3-1*:* Regression model results ^a^

|  | | | | |
| --- | --- | --- | --- | --- |
|  | P value | aOR | 95% C.I.for aOR | |
|  |  |  | Lower | Upper |
| Age (years) | **.027** | 1.130 | 1.014 | 1.258 |
| rPATD appropriateness score | **.022** | .452 | .229 | .892 |
| rPATD concerns about stopping score | **.036** | .483 | .245 | .953 |
| Chronic pulmonary disease | .062 | .242 | .054 | 1.077 |
| Gastric disease ^b^ | **.011** | **5.217** | **1.452** | **18.740** |
| Prostatic disease ^c^ | .**016** | .217 | .063 | .755 |
| Constant | .521 | .069 |  |  |

Abbreviations: aOR, adjusted odds ratio; CI, confidence interval; rPATD, revised Patients’ Attitudes Towards Deprescribing.

1. Multiple logistic binary regression with backward LR method. The rPATD global question "*If my doctor said it was possible, I would be willing to stop one or more of my regular medicines*" was dichotomized to a binary outcome *agree* (strongly agree or agree) and *disagree* (unsure, disagree, or strongly disagree), designated as *“willingness to have medications deprescribed.”* The patients’ demographic and clinic characteristics, the independent variables, with p < 0,100 bivariate analysis, were selected to be included in the multiple binary logistic regression model. The variable *Age* was considered relevant and was also included. All assumptions were satisfied for the selected variables, except the rPATD *appropriateness* score, but it was included in the multiple binary regression because it was considered a clinically relevant variable. As a result, the independent variables included were Age (years), Sex, Medical Appointments (last 12 months), rPATD Burden score, rPATD Appropriateness score, rPATD Concerns about stopping medications score, chronic pulmonary disease, gastric disease, prostate disease, chronic pain, neurologic disease. N=192 and 160 were included in the analysis.
2. any history of ulcer disease treatment or prevention, ulcer bleeding, or GERD (gastroesophageal reflux disease)
3. prostate benign hypertrophy, prostatic neoplasia

This model was significant [X2 (8) = 39.091; p<0.001], explaining 37.4 % of the variance (Nagelkerke R Square) and correctly predicted 87.5 % of the results. There was a good model fit (Hosmer-Lemeshow test nonsignificant, p=0.834).

The leverage cut-off point was [3(11 + 1/*160*)] = 0.225.

Table A3-2: Casewise list^a^

| Case | Selected Status^b^ | Observed | Predicted | Predicted Group | Temporary Variable | | |
| --- | --- | --- | --- | --- | --- | --- | --- |
|  |  |  |  |  | Resid | ZResid | SResid |
| 1 | S | d** | .986 | a | -.986 | -8.323 | -2.934 |
| 20 | S | d** | .837 | a | -.837 | -2.267 | -2.020 |
| 22 | S | d** | .976 | a | -.976 | -6.397 | -2.751 |
| 24 | S | d** | .930 | a | -.930 | -3.653 | -2.325 |
| 28 | S | d** | .951 | a | -.951 | -4.416 | -2.478 |
| 29 | S | d** | .940 | a | -.940 | -3.959 | -2.413 |
| 30 | S | d** | .903 | a | -.903 | -3.049 | -2.192 |
| 35 | S | d** | .901 | a | -.901 | -3.025 | -2.188 |
| 36 | S | d** | .901 | a | -.901 | -3.025 | -2.188 |
| 45 | S | d** | .871 | a | -.871 | -2.601 | -2.152 |
| a. Cases with studentized residuals greater than 2.000 are listed. | | | | | | | |
| b. S = Selected, U = Unselected cases, and ** = Misclassified cases. | | | | | | | |
